# A Qualitative Exploration of Facilitators and Barriers to Physical Activity Participation among Chinese Retired Adults in Hong Kong

**DOI:** 10.3390/ijerph19063495

**Published:** 2022-03-16

**Authors:** Ying Huang, Oi-Lam Ng, Amy S. C. Ha

**Affiliations:** 1 Department of Sports Science and Physical Education, Faculty of Education, The Chinese University of Hong Kong, Hong Kong 999077, China; yinghuang@link.cuhk.edu.hk; 2 Department of Curriculum and Instruction, Faculty of Education, The Chinese University of Hong Kong, Hong Kong 999077, China; oilamn@cuhk.edu.hk

**Keywords:** physical activity, retired adults, facilitators, barriers, interviews

## Abstract

The purpose of this study was to identify facilitators and barriers associated with physical activity participation among retired Chinese adults in Hong Kong. This study adopts an interview research design in order to generate an in-depth understanding and insights into the participants’ thoughts, motivators and experiences of physical activity participation. Independent, semi-structured interviews with 10 retired participants (aged 54–74) were conducted based on an interview protocol with open-ended questions prompting the participants to describe their experiences. Transcribed texts were analysed using thematic analysis, combining both deductive and inductive analysis techniques. Common physical activities reported were walking, stretching exercise and jogging/running. Participants responded that their physical activity level increased since their retirement. We report the interview results according to the themes which emerged from the analysis: (1) physical and mental health, (2) socio-emotional factors, (3) environmental context, (4) family responsibilities. We found that the themes (1), (2) and (3) act either as a facilitator or a barrier for the participants interviewed, while theme (4) family responsibilities act as a barrier. The findings suggested that future physical activity interventions for retired Chinese adults should include more physical activity knowledge, such as the benefits to physical and mental health brought by physical activity and social elements, considering the specific challenges that participants are confronted with (from the family side).

## 1. Introduction

There is a continuous ageing trend in the world, with the percentage of adults aged 65 and above projected to rise from 9% in 2019 to 16% by the year 2050 [1]. The increase in life expectancy induces an increasing expenditure on health care costs, as the prevalence of chronic disease rises with age [2]. The benefits of physical activity participation have been well documented [3,4]. People who engage in sufficient physical activity had a 31% reduction in mortality compared with people that are physically inactive [5]. Insufficient physical activity is the fourth leading risk factor for global mortality and noncommunicable diseases such as cardiovascular disease, cancer and diabetes [6]. To prevent and delay the development of chronic diseases, adults should adopt a healthy lifestyle with sufficient physical activity [7]. 

It has been well accepted that major life events (i.e., transition to university, beginning to work, marriage, pregnancy) have a strong effect on physical activity [8,9,10]. For mid-to-old-age adults, life events such as retirement increase physical activity, while long-term trajectories across retirement decrease physical activity [11]. More specifically, life events in later life, such as moving into an institution (i.e., nursing home) and interpersonal loss, decrease physical activity, while long-term widowhood increase physical activity [12]. Retirement is an important turning point in a mid-to-old adult’s life. Retirement offers opportunities to develop a different lifestyle, as the prolonged time previously spent working can be spent on other self-selected activities, and new habits can be developed. Thus, it is worthwhile to implement and investigate the intervention of retired people, with an aim to preventing mid-to-old-age adults from lapsing into an inactive lifestyle [13]. 

The results from previous systematic reviews demonstrated that interventions conducted among older people were found effective in increasing the physical activity level and functional ability, typically at 12 months [14,15], but long-term effectiveness (i.e., 24 months) was unknown [16]. It has been recognized that a deeper understanding of what the facilitators and barriers are to older adults would enable the development of interventions that acknowledge and address the physical, psychological and social factors that influence their participation in the long term [17]; in particular, a number of qualitative studies were implemented to answer such questions [10,18,19,20,21,22]. Previous systematic reviews [23] of qualitative research indicated that physical activity can help retired adults to regain a feeling of purpose (being needed in collective activities) and structure the day (by creating daily routines), while the building of self-esteem can be a challenge. A previous systematic review [24] also identified six themes of older people’s perspectives on physical activity participation: social influences, physical limitations, competing priorities, access difficulties, personal benefits and motivation and beliefs. Previous research also focusing on specific ethnic groups [22,25,26,27], different themes such as cultural and structural barriers, gender norms and expectations as barriers, faith and education as facilitators also emerged. 

According to a previous study [7] investigating worldwide trends in insufficient physical activity participation and the national surveys in China [28], even though the prevalence of insufficient activity was stable (27.5%), the leisure time spent on physical activity has increased in China since 2000. Despite the increased participation in physical activity, the survey also observed the worsened physical fitness level and the increased prevalence of overweight and obesity. Thus, effective interventions for physical activity promotion are needed to reduce obesity and improve physical fitness in the Chinese population. Among the Chinese population, although the number of physical activity studies has increased in recent years, they are still limited compared with those conducted in the western population [29,30]. As previous studies suggested, it is important for intervention studies that aimed at increasing physical activity levels to involve tailored contents to suit local opportunities and the environment [15,16]. For example, the insights from an observational study [31] in China showed that Chinese park uses were more active than studies carried out in the United States, so that the interventions developed to promote active parks would be a possible effective intervention among the Chinese population. Additionally, taking care of grandchildren was reported as an uniquely significant barrier to physical activity participation in the Chinese population [32]. Thus, more evidence on these and other (e.g., culture and environment) determinants of physical activity during retirement in the Chinese community is needed. 

The overall objective of the current study is to understand the facilitators and barriers to physical activity participation for retired Chinese adults. Furthermore, the study aims to examine the various facilitators and barriers affecting retired Chinese adults with different physical activity levels (i.e., low, middle, high) in order to shed light on why certain adults are more (or less) physically active than others. Specifically, the research questions posed are: (1) What factors served as facilitators or barriers to physical activity participation among Chinese retired adults in Hong Kong? (2) How do these factors compare in terms of their impact on the physical activity participation of Chinese retired adults in Hong Kong with different physical activity levels?

## 2. Materials and Methods

### 2.1. Research Design

This study adopts an interview research design in order to generate an in-depth understanding and insights into the participants’ thoughts, motivations and experiences of physical activity participation (or lack thereof). According to Turner III [33] (p. 754), interview designs “can be developed to obtain thick, rich data utilizing a qualitative investigational perspective” [34]. This is because interviews provide in-depth information pertaining to participants’ experiences and viewpoints around a particular subject or matter. In particular, a semi-structured interview method was utilized to generate rich and thick data, at the same time enabling the researchers to effectively extract similar themes or codes from the interview transcripts. The semi-structured interviews were conducted based on an interview protocol with open-ended questions prompting the participants to describe their experiences. Sample questions from the interviews are shown in Table 1. 

### 2.2. Participants

Aligned with the interview research design, a low number of participants were recruited to ensure the in-depth examination of the data obtained. Given that Creswell [34] has emphasized the importance of selecting appropriate interview candidates by the method of criterion-based sampling, this study recruited participants aged 50 or older; retired or without full-time or part-time jobs; fluent in Chinese (both Cantonese or Putonghua); physically mobile; healthy or having common chronic diseases but ambulatory; without a cognitive disability and able to give written consent. 

A purposive sampling approach was used, in which ten individuals were recruited out of a sample of 153 people who participated in the baseline measurement of a cross-sectional study (February 2021–May 2021) examining the sample’s participation in daily and physical activities. From the cross-sectional study, we contacted 30 participants to obtain reasonable representations of participants from each group of physical activity level. In particular, since there was a limited number, i.e., 12 male participants, from the cross-sectional study who were determined to be in low and moderate in physical activity levels, we contacted all 12 of them, but only one of them accepted the invitation to participate in the study. The invitations were sent twice through WhatsApp (5 days apart between the invitation and reminder invitation), and the participants who have not replied were considered as having refused to participate. As shown in Table 2, the average age of the 10 participants was 63.8 years, with 2 male participants and 8 female participants, and the recruitment team contacted the same number of female and male participants. Table 2 also summarizes the participants’ demographic information as collected from the baseline questionnaire. The physical activity level was measured by an international physical activity questionnaire—short form (IPAQ-SF) [35], and the reliability and validity of the Chinese version of IPAQ-SF was reported by a previous research [36].

All participants provided an informed consent to participate, including the permission to be audiotaped during the interview. All participants received a small incentive for their participation. Ethical approval for the study was obtained from the Survey and Behavioural Research Ethics of the Chinese University of Hong Kong. The interviews took place in a quiet conference room in the authors’ institution and lasted between 1 and 1.5 h from 22 October to 5 November 2021. 

### 2.3. Analyses 

All interviews were anonymized and transcribed verbatim by trained student assistants. Once transcribed, the interview transcripts were read through, and the accuracy was checked by the researcher. A thematic analysis method [37] was used to analyse the data, combining both deductive and inductive analysis techniques, in order to generate plausible themes based on excess evidence and the lack of counter examples [38]. That is, an analytic framework was constructed based on the major relevant themes to the interview protocol through deductive analysis. After that, an inductive analysis process was used to capture potential additional themes not captured in the previous deductive analyses. The method of constant comparative analysis [39], which included continually sorting through the texts, identifying patterns, categorizing the data collected, coding the information and refining the patterns through a comparison across all categories, was used throughout the analysis stage. A thematic analysis coupled with the use of constant comparative strategies [40] is appropriate in qualitative studies because it facilitates data analysis according to commonalities, relationships and differences [41]. These steps, coupled with the chosen sampling, enable the generation of a theory about physical activity participation by retired adults with different physical levels, that is integrated and close to the data.

To elaborate, the analysis took on the following steps. First, the researchers reviewed the data in their entirety. Then, common patterns were organized into initial, tentative themes, namely, facilitators and barriers to participants’ physical activity participation. The process was conducted and recorded in Microsoft Excel. At this stage, a triangulation of data was performed by seeking supporting (or non-supporting) evidence within individual responses across different interview times. The researchers went over the data a second time to explore the degree to which general patterns matched the data. During this time, rich discussions were held among the researchers about the data and emerging themes until a consensus was reached. Once no new themes emerged, it was assumed that data saturation had been reached and that the final themes describing retired Chinese adults’ physical activity participation had been generated.

### 2.4. Trustworthiness

To establish trustworthiness, the current study followed the guidelines of basic qualitative research [42], as well as those of coding and thematic analysis [43]. First, we provided detailed selected transcriptions from participants and triangulated data from different parts of the interviews to ensure the credibility of the results. Second, thick descriptions with concrete details were discussed and analysed to delve into any tacit knowledge, such as one’s culture and values, that might enhance the interpretation of results. Third, the study’s initial findings were sent to the participants for member reflections; this provided opportunities for the participants to raise questions, critique and feedback, i.e., member checking [44]. Moreover, participants with different physical activity levels (i.e., low, moderate and high) were selected to achieve transferability, referring to the generalizability of the inquiry on a case-to-case basis [45]. To achieve dependability, we used an interview guide and held discussions among the researchers to ensure the research process was logical, traceable and clearly documented [45]. Finally, confirmability was achieved by keeping an audit trail for the research process, by recording and transcribing all conducted interviews as well as keeping a record of the coding process. 

## 3. Results

We report the interview results according to the themes which emerged from the analysis: (1) physical and mental health, (2) socio-emotional factors, (3) environmental context and (4) family responsibilities. We found that the themes (1), (2) and (3) act either as facilitators or barriers for the participants interviewed, while (4) family responsibilities act as a barrier. 

Overall, nine out of ten adult participants agreed that they did not have regular physical activity habits before retirement or were about to retire because of work. Upon retirement, they expressed that they had more time to participate in physical activity. Moreover, they mainly reported three types of physical activities that they regularly participate in, namely walking, running and stretching. Generally, we found that all participants were unaware of the current World Health Organization’s physical activity guidelines (minimum 150 min moderate physical activity/week, strength and balance 3 times/week) which implies the lack of knowledge of physical activity:

“*I heard someone say that every day people need to walk 10,000 steps, but others said 10,000 steps are too much, I think I may not meet 10,000 steps and maybe 6000–8000 steps daily*.”

(Participant E, 60 years old, moderate physical activity level)

Participants reported that they received information about their age-appropriate physical activity from the media and the internet and through direct contact with friends and families. In terms of resources of physical activity online, different discussions were stated:

“*I think our government should do more promotions in physical activities in the local community, the simplest way would be promoted through social media such as newspapers, TV.*”

(Participant C, 64 years old, moderate physical activity level)

“*There are too many sources online for physical activity, all of my friends keep several links for physical activity videos, and we would send to each other, the problem is how to keep doing it.*”

(Participant D, 75 years old, moderate physical activity level)

As mentioned above, participants who were familiar with the internet mentioned that there is too much information online. Furthermore, those who may not be familiar with the internet reported a lack of information related to physical activity in the local community.

### 3.1. Physical and Mental Health

All participants recognized that physical activity is a physical need for health at their age, regardless of their physical activity level:

“*When I was young, I did it [playing basketball] for the feeling of being competitive and excitement, but now, it’s for health.*”

(Participant B, 74 years old, low physical activity level)

“*I have too much time after retirement, I do not know how to make use of it. I do not know how much physical activity is enough, but I know doing exercises are beneficial to my health, and so I am doing it. I am afraid that if one day I could not walk in the future, then I would feel guilty for doing [physical activity] too late and having too many excuses for not doing physical activities in the past.*”

(Participant C, 64 years old, moderate physical activity level)

“*Although I am not enthusiastic about doing physical activities, it is definitely a need for health, so now, I am doing it if I have time.*”

(Participant J, 61 years old, high physical activity level)

As shown above, there was a consensus among the participants that regardless of their current level of physical activity, they agree that physical activities would bring certain benefits to their health. For example, for Participant B (with a low level of physical activity), “health” was the primary reason and served as an intrinsic motivation for her to participate in physical activity.

Furthermore, the participants’ attitude towards physical activity participation changed when they retired. When working, physical activity participation to them means a way to socialize and release working pressure, and when retired, they perceived physical and mental benefits as the primary facilitator for physical activity participation, as evident in the following transcript:

“*I used to go fishing or diving with my friends when I was young, the activity was totally for socialization, but now (doing physical activities) for health only.*”

(Participant G, 54 years old, moderate physical activity level)

“*In the past, doing physical activity was a kind of way to realise working pressure for me, when retired, no working pressure anymore, the motivation of health increased when participating in physical activities.*”

(Participant E, 60 years old, moderate physical activity level)

Often, the participants were made aware of their need to be active in order to stay healthy. They were informed by either their friends, to whom they could relate, or the doctor, who served as authority figures:

“*The loss of one of my best friends because of cancer alarmed me to pay more attention to my health…because of her, I knew it was time to take care of myself. And then, we, a group of friends joined the Charity Cancer Run that year, and then the following years, my families joined…*”

(Participant G, 54 years old, moderate physical activity level)

“*Doctors often suggest us (husband and I) do physical activity regularly to improve hypertension, hyperglycemia and hyperlipidemia, although the process was tough, after that, I feel relaxed.*”

(Participant A, 57 years old, low physical activity level)

As mentioned, being aware of their current physical condition could serve as a facilitator in their participation in physical activities:

“*The first day I ran, the running process was painful already, and then the second day I must feel pain and then I would struggle, should I run today or rest?*”

(Participant A, 57 years old, low physical activity level)

“*I was in the hospital many times because of heart failure, I cannot do high intensity physical activity, so I play Taiji.*”

(Participant D, 75 years old, moderate physical activity)

“*I fell twice in the year 2019 fracturing one of my toes and one of my fingers, although I am still joining the exercise classes, I am very careful and would not do activities with high intensity.*”

(Participant G, 54 years old, moderate physical activity level)

“*I do not think my physical condition has an influence on my physical activity participation, for example, I feel the functional ability of my legs declined, my fitness level and the performance declined, it easier to fear fatigue than before, I think the decline is hard to control by myself.*”

(Participant H, 67 years old, high physical activity level)

The above four participants reported their current physical conditions, such as cardiovascular disease (i.e., heart attack), physical pain (i.e., joint and muscle) and previous injuries; three of them thought their current physical condition would influence their physical activity participation. Comparing their physical activity level, it was observed that Participant A, with a low level of physical activity, reported that her physical condition (pain) would have a direct influence on her physical activity participation. On the other hand, Participant D and G, with moderate physical activity, only mentioned that their current physical condition would be a barrier to participate when doing high-level physical activities, while Participant H, with a high physical activity level, did not think that his current physical condition would influence his physical activity participation. 

### 3.2. Social-Emotional Factors

People in three different physical activity levels all reported that *social interaction with others* was a significant facilitator to their participation. This was demonstrated by the following responses by the participants (a detailed analysis will follow):

“*If I see my daughter doing yoga or stretching at home, I will join her; sometimes, one of my friends teaches stretching in the community centre, and I would join her.*”

(Participant A, 57 years old, low physical activity level)

“*If one of my families, such as my son, daughter-in-law, the grandson who propose hiking, then we would go there together. Because if we have time hiking together, quite different from eating together which is so normal, it looks like we have the same goal which would make us feel closer.*”

(Participant B, 74 years old, low physical activity level)

“*I play badminton twice a week with my husband and our friends, when we have four people available, my husband would get up earlier to book the sports centers, I was not that into badminton before retirement maybe 2–3 months once, but now playing badminton means meeting friends and we always eating together afterwards which become a routine in my daily life.*”

(Participant F, 59 years old, moderate physical activity level)

“*I believed that the influence of friends in physical activity participation is important to me. I joined the stretching class every Monday and the dancing class every Thursday with my friends. At the very beginning, she dropped the class because of the COVID-19 and after a few months she joined again, I feel quite different if I joined alone or with her. Sometimes our coach [Name] would make fun of her and I would join...Actually joining the exercise classes, the first 60 min of dancing are already very tiring, and the remaining 30 min have to be overcome, I know I cannot give up halfway.*”

(Participant G, 54 years old moderate physical activity level)

“*I joined the yoga class invited by my daughter, and then we can chat during the round trip; if hiking, I would go with my husband and of course we chat. Exercise alone is really hard to persist, but if I joined the scheduled exercises class, I would remind myself go to each class at the said time.*”

(Participant E, 60 years old, moderate physical activity level)

“*Running is a lonely exercise, yes, of course, I can run alone, but if running with my friends, and then we can have lunch together, I will feel satisfied, but conversely, if we leave and go home alone immediately after running, I do have a feeling of loss. I think the most important thing for us (friends) to run together is we have the same goals, and we are in similar fitness level.*”

(Participant H, 67 years old, high physical activity level)

In summary, the social interaction that accompanied physical activities was mentioned by eight of ten participants to be more important than the physical activities themselves. Examining the above quotes more closely, social interactions occurred before (e.g., making appointments with others), during (e.g., especially among people with the same intensity) and after the physical activity (e.g., eating together). These social interactions became a necessary part of physical activities for those who claimed to enjoy them. On the contrary, two of three participants with high physical activity levels reported enjoying doing exercise alone, indicating that they did not enjoy the social aspects of the physical activity:

“*I did not like walking with my friends and colleagues because when people walked together always talked and stopped to take photos.*”

(Participant J, 61 years old, high physical activity level)

“*I do not need others to join me when I am doing physical activities. I can arrange my time freely for example after lunch or dinner. I like walking at my speed, so if I walk alone, I do not need to care about others and concern their walking speed…Actually, I want to walk with my son but he has others and we walk at different speeds.*”

(Participant I, 67 years old, high physical activity level)

When comparing their physical activity levels, the following observations were made: Participants with low or moderate physical activity levels tend to join different kinds of activity classes and always participate with their families or friends (i.e., yoga, dancing, stretching); participants with high physical activity tend to enjoy doing physical activities alone. Participants J and I, both with a high physical activity level, mentioned that they did not enjoy the social aspects of physical activity due to the different intensity (i.e., walking speed) and different time arrangements with their physical activity partners. Although Participant H, also with a high physical activity level, enjoyed the social interaction with others during the physical activity, he pointed out that it required that his physical activity partner needed to have the same fitness level as him. These differences may be explained by their physical activity level, as participants with low or moderate physical activity levels may not necessarily enjoy physical activity but felt motivated to engage in it by feeling a sense of relatedness from doing so. On the other hand, Participants J and I, with a high physical activity level, may prefer to walk alone because of their high engagement in the physical activity (i.e., walking) and their perceived distraction when doing so with friends and colleagues.

Interestingly, Participant I mentioned that although the walking speed with her son is also different, she is still willing to participate in physical activity with her son. Likewise, Participants A and B, both with a low physical activity level, also mentioned that the company of families would initiate their physical activity participation; these results are consistent with the previous systematic review of social support and physical activity [46]. 

The structured exercise class was mentioned as the facilitator by two participants with a moderate physical activity level (Participants E and G) and one participant with a low physical activity level (Participant A). These participants expressed that the scheduled classes would facilitate their physical activity participation in two ways: having a fixed time or place for physical activity would remind them to participate in physical activity regularly, maintaining the time and quantity of physical activity. 

On the other hand, Participant B mentioned that when he retired, the reduction of his ”social circle”—lack of exercise partners (the previous colleagues) and the time conflict with his wife, who was still working, were a barrier to his physical activity participation:

“*I enjoyed playing basketball with my friends and joining competitions, but now I retired while they still working, so it is hard to meet and play again. Because my wife has not retired yet, if we go hiking, I need to wait until she has holiday.*”

(Participant B, 74 years old, low physical activity level)

As shown in the above transcripts, various social activities consistently accompanied participants’ physical activities. This suggests that physical activities for retired adults may be connected to socio-emotional aspects of their retired lifestyles. 

### 3.3. Environmental Context 

We observed two kinds of environment-related factors mentioned by the participants: factors related to the weather condition and a series of social-distancing regulations implemented under COVID-19:

“*During the COVID-19, I would never go outside to do physical activities.*”

(Participant A, 57 years old, low physical activity level)

“*At the very beginning, all government sports facilitators closed, we did not have a place to play badminton, so we stopped…and then facilitators re-open, we wore masks play badminton…*”

(Participant F, 59 years old, moderate physical activity level)

“*Although there are still some limitations in using the local sport facilitates in terms of the number of people, the re-open of the sports centers make sure at least I have a place to doing exercise regular……On the other hand, I think only the hot weather and the health condition would be barriers to my physical activity participation.*”

(Participant C, 64 years old, moderate physical activity level)

“*I think the weather do help, but good weather may not have a chance to do exercise. For example, I would ask my friends if they were available for hiking if the weather is good one day, but they may engage…*”

(Participant G, 54 years old, moderate physical activity level)

“*The process during the Taiji is enjoyable, it provides me with an opportunity to feel my body and the movements, especially when I heard birdsong, see the green trees, blue sky, and clouds.*”

(Participant D, 74 years old, moderate physical activity level)

“*Even during the COVID-19, I kept doing exercise because there is a small garden inside my housing estate, and I can walk there every day. But I think the weather if raining or typhoon would be a barrier to me for the participation.*”

(Participant I, 67 years old, high physical activity level)

“*I have a fixed route from my home to [destination] and I like walking there, the views are good, and the air is fresh. The problem is the weather, if too hot, it definitely not suitable for walking, I would feel uncomfortable.*”

(Participant J, 61 years old, high physical activity level)

The above quotes suggest that environmental facilitators towards physical activity include having a fixed place or route for doing exercise (e.g., sports centre) and good weather conditions. When talking about the influence of COVID-19 on physical activity, people with different physical activity levels also showed different attitudes. Participant A (low physical activity level) believed it was very challenging to go outside, while participants with a moderate physical activity level thought the influence of COVID-19 was acceptable, and participants with a high physical activity level mentioned they were not influenced. As mentioned above, even participants with a high physical activity level stated that the bad weather conditions were a barrier to their physical activity participation, which manifested itself most among participants who used to do physical activities outside their residence. The possibility of doing exercise at home has not been mentioned.

### 3.4. Family Responsibilities

Some participants reported that family responsibilities such as providing grandchild care or doing housework were the barriers to physical activity participation:

“*I love doing exercise, I always played basketball with my colleague when I still working. But now I need to take care of my grandson, so I do not have time to do it, even now he is in secondary school, you know owing to the current COVID-19, he just needs to go to school half-day, so I still need to take care of him because other families still working.*”

(Participant B, 74 years old, low physical activity level)

“*Actually, I only have time to do physical activity recently, at the early retirement stage, I need to take care of my grandsons but now my body gets worse, so I do not need to take care of them anymore…*”

(Participant D, 75 years old, moderate physical activity level)

“*Although I have one domestic helper in my house, there is still much housework for me to do and arrange so physical activity is only a small part of my life.*”

(Participant J, 61 years old, high physical activity level)

The common belief for the public was that retirement is associated with more free time, that participants could have plenty of time to participate in physical activities if they wanted. However, the participants above mentioned that their dedication to the family (e.g., taking care of grandchildren) after retirement made them have no time to participate in physical activities. Even for Participant B (low physical activity level), who was active during leisure time before retirement, stopped doing physical activities to take care of his grandson.

## 4. Discussion

The overall objective of the study is to extend the current research by gaining further insights into facilitators and barriers to physical activity participation for retired Chinese adults. Furthermore, the study examined the various facilitators and barriers affecting retired Chinese adults with different physical activity levels (i.e., low, middle, high) in order to shed light on why certain adults are more (or less) physically active than others. Consistent with ecological models [47], intrapersonal (physical and mental health), interpersonal (socio-emotional factors, family responsibilities) and environmental determinants were stated in the current study. More specially, we found that among retired Chinese adults, physical and mental health, socio-emotional factors and environmental context act either as facilitators or barriers; family responsibilities were a special barrier to their physical activity participation. The reported facilitators and barriers to physical activity participation in this study were strongly dependent on the individuals’ current physical activity level. Obviously, participants with a low physical activity level are more likely be influenced by barriers such as the environmental factors. Participants with low or moderate physical activity levels and barriers such as their current physical condition, a lack of social company, the influence of COVID-19 and taking care of their families would have direct influence on their decision of engaging in physical activity or not; the socio-emotional factors, especially the company of their families, would be more likely to initiate their physical activity participation. This study also identified that retirement, as an important life event, is also a determinant of physical activity participation in Chinese adults. The majority of people in the current study had an increase in the physical activity level after retirement.

First of all, we found that participants with low, moderate and high physical activity levels, and even those who participated in exercise classes, still lack the knowledge of physical activity guidelines. Thus, future interventions could include an easy understanding and following of ‘educational’ sessions with an overall introduction to the physical activity guidelines for retired adults. A previous review indicated that three out of nine interventions including physical activity education or counselling sessions showed a positive effect when reporting long-term results [48]. More importantly, efforts should be made to guide older adults to convert the knowledge into concrete actions and engage in sufficient and effective physical activity in their daily life, such as adopting behaviour change techniques based on the participants’ characteristics [49].

Our findings showed different views towards physical activity resources among participants. Adults who are familiar with the internet can find enough information if they want to, while others reported that the resources are limited. Not all older adults have access to the internet (the gap was called the “grey digital divide”), and previous literature describes numerous factors (i.e., costs, physical constraints, cognitive impairments) explaining this gap; a strong predictor for internet use was the encouragement by the family and friends [50]. Compared with traditional professional-led interventions (i.e., advice from doctors), interventions adopting technology such as mobile health apps may provide an alternative approach during the current pandemic. The previous meta-analysis found that interventions that adopted technology (i.e., mobile apps) were found to be effective in increasing steps (506 steps) among older adults [51]. Recently, older adults’ internet access and technology skills have also been discussed [52] for promoting physical activity during COVID-19; however, the article highlighted the role of governments and non-profit agencies but has not addressed the importance of the family and friends for each individual. Thus, we believe that the support from families or friends for interventions adopting a technological approach aimed at increasing physical activity among retired adults would be necessary.

Interestingly, we found that the social interactions that accompany physical activity may be more important than the physical activities themselves, as those socio-emotional factors became a necessary part of the physical activities for the participants in the current research. Previous studies reported that social support for physical activity was associated with higher levels of physical activity among older adults, specifically when supported by their families [46,53,54]. For retired adults with low physical activity levels, the results in the current study suggested that the company of family members is more likely to initiate their participation. The participants in the current qualitative study also provided insights indicating that there are different types of social support for physical activity participation, with the interactions including the periods before (i.e., making appointments), during (i.e., talking) and after (i.e., participating in other activities) the physical activity participation. Physical activity is sometimes an excuse for a gathering, and what they did after physical activity seems more important and attractive to them. Thus, future intervention studies may add more “after physical activity” sessions to attract participation. 

A mentioned barrier to the participation in physical activity was family-related factors and taking care of grandchildren, which was considered a priority. There is an increase in both the incidence and prevalence of grandparental care for grandchildren [55]. A previous study found that grandparents who cared for their grandchildren reported a better mental and physical health compared with those who do not [56], and such activities may help them maintain their cognitive functions by stimulating the brain [57]. This finding suggested that efforts could be made to relate physical activity to the specific challenges that participants were confronted with, and the support from family members would be necessary for this population. Interventions that adopted a family-based approach are effective in increasing children’s physical activity [58], and the attempt has also been made in the local context [59], in the parents and children dyads. Thus, future interventions could also consider the adoption of a family-based approach to increase retired adults’ physical activity.

Interestingly, the commonly reported environmental barriers (i.e., traffic safety, sidewalks, distance and price of the sports centres) in the same age group in the western countries were not mentioned in the current population [60,61,62,63]. The mentioned environmental barriers were related to COVID-19 and the hot weather. The main reason would be the environmental variation by country. Hong Kong was considered as the most “activity-friendly” built environment among 11 cities around the world [64], with a sidewalk availability at 97% on most streets and a high availability on transit stops within 10–15 min from home and recreation facilities. These findings confirm the importance of designing intervention content that suits the cultural difference and the local context. 

The present study has some limitations that need to be acknowledged. First, the findings from the qualitative study only refer to ten participants. Participants with a low physical activity level were hard to approach and recruit, and thus the number of participants with a low physical activity level is lower than that of participants with moderate to high physical levels. Hence, we cannot generalize our findings to other populations. Secondly, although we have tried to approach people with different physical activity levels (low, moderate and high) by using the IPAQ-SF [35], the physical activity levels were self-reported. We do not know the actual behaviour and level of physical activities of our participants, which could have biased the findings. 

## 5. Conclusions

The findings from this study are important for better understanding mid-to-old-age retired Chinese adults’ facilitators and barriers. Future interventions could introduce more knowledge related to physical activity, such as the benefits of physical activity to mental health. Considering the socio-emotional factors facilitating retired adults’ physical activity participation, such as scheduled activity classes with friends or families, can help design meaningful “after physical activity” sessions. For the participants with a low level of physical activity, inviting family members may encourage and initiate their participation. Interventions could also consider the specific challenges that participants are confronted with (from the family side).

## Figures and Tables

**Table 1:** Sample questions from the interviews.

Questions
1. Tell me about the experience of your physical activity participation.
2. Do you think you need to participate in physical activity? Why or why not?
3. What are the benefits that you believe physical activity would bring to you?
4. What are the barriers for your physical activity participation?
5. What are the facilitators for your physical activity participation?
6. Tell me whether you enjoyed the experience of physical activity participation.
7. Tell me whether you dislike the experience of physical activity participation.
8. What do the people around you think about your physical activity?

**Table 2:** Participants’ demographic information.

Participant ID	Physical Activity Level	Age	Gender	Marital Status
A	Low	57	Female	Married
B	Low	74	Male	Married
C	Moderate	64	Female	Married
D	Moderate	75	Female	Divorced
E	Moderate	60	Female	Married
F	Moderate	59	Female	Married
G	Moderate	54	Female	Single
H	High	67	Male	Married
I	High	67	Female	Widowed
J	High	61	Female	Married
